# Doxorubicin Induces Endotheliotoxicity and Mitochondrial Dysfunction *via* ROS/eNOS/NO Pathway

**DOI:** 10.3389/fphar.2019.01531

**Published:** 2020-01-10

**Authors:** Huan He, Liang Wang, Yang Qiao, Qing Zhou, Hongwei Li, Shuping Chen, Dong Yin, Qing Huang, Ming He

**Affiliations:** ^1^ Jiangxi Provincial Institute of Hypertension, The First Affiliated Hospital of Nanchang University, Nanchang, China; ^2^ Jiangxi Provincial Key Laboratory of Basic Pharmacology, Nanchang University School of Pharmaceutical Science, Nanchang, China; ^3^ Department of Rehabilitation, The First Affiliated Hospital of Nanchang University, Nanchang, China; ^4^ Jiangxi Provincial Key Laboratory of Molecular Medicine, The Second Affiliated Hospital of Nanchang University, Nanchang, China; ^5^ Jiangxi Provincial Institute of Cardiovascular Diseases, Jiangxi Provincial People’s Hospital Affiliated to Nanchang University, Nanchang, China

**Keywords:** doxorubicin, vascular endothelium, endotheliotoxicity, mitochondria, ROS/eNOS/NO pathway

## Abstract

**Background:** Doxorubicin (Dox) can induce endotheliotoxicity and damage the vascular endothelium (VE). The most principle mechanism might be excess reactive oxygen species (ROS) generation. Nevertheless, the characteristics of ROS generation, downstream mechanisms, and target organelles in Dox-induced endotheliotoxicity have yet to be elucidated.

**Methods and Results:** In order to explore the related problems, the VE injury models were established in mice and human umbilical vein endothelial cells (HUVECs) by Dox-induced endotheliotoxicity. Results showed that the activities of lactate dehydrogenase (LDH) and creatine kinase of mice’s serum increased after injected Dox. The thoracic aortic strips’ endothelium-dependent dilation was significantly impaired, seen noticeable inflammatory changes, and brown TUNEL-positive staining in microscopy. After Dox-treated, HUVECs viability lowered, LDH and caspase-3 activities, and apoptotic cells increased. Both intracellular/mitochondrial ROS generation significantly increased, and intracellular ROS generation lagged behind mitochondria. HUVECs treated with Dox plus ciclosporin A (CsA) could basically terminate ROS burst, but plus edaravone (Eda) could only delay or inhibit, but could not completely cancel ROS burst. Meanwhile, the expression of endothelial nitric oxide synthase (eNOS) decreased, especially phosphorylation of eNOS significantly. Then nitric oxide content decreased, the mitochondrial function was impaired, mitochondrial membrane potential (MMP) impeded, mitochondrial swelled, mitochondrial permeability transition pore (mPTP) was opened, and cytochrome C was released from mitochondria into the cytosol.

**Conclusion:** Dox produces excess ROS in the mitochondria, thereby weakens the MMP, opens mPTP, activates the ROS-induced ROS release mechanism, induces ROS burst, and leads to mitochondrial dysfunction, which in turn damages VE. Therefore, interrupting any step of the cycles, as mentioned above can end the related vicious cycle and prevent the occurrence and development of injury.

## Introduction

Since its advent in the 1960s, Doxorubicin (Dox) has been widely used in the treatment of various malignant tumors because of its broad spectrum and high efficiency (Bonadonna et al., 1969). However, due to its dose-dependent cardiotoxicity, its clinical application is greatly restricted (Singal and LIiskovic, 1998; Vejpongsa et al., 2014). In the past two decades, the toxicity of Dox to blood vessels, especially to vascular endothelium (VE) has gradually attracted people’s attention (Kotamraju et al., 2000; Wojcik et al., 2015). As critical components of the cardiovascular system, the integrity of VE structure and function is extremely important, but it is vulnerable to adverse physical, chemical, or biological stimulation and damage, leading to toxic side effects, which directly affects the cardiac function (Soultati et al., 2012; Wojcik et al., 2015). It has been noted that the toxicity of Dox to the myocardium and VE is often accompanied by and may even cause and affect each other (Soultati et al., 2012; Wojcik et al., 2015; Cappetta et al., 2018).

Many studies have found that there are various reasons for Dox’s cardiotoxicity or endotheliotoxicity (Wojcik et al., 2015; Cappetta et al., 2018; Renu et al., 2018), however, a common factor is that Dox itself may induce oxidative stress, resulting in excessive reactive oxygen species (ROS) generation (Wolf and Baynes, 2006; Octavia et al., 2012; Angsutararux et al., 2015; Cappetta et al., 2017). In previous studies, we showed that Dox toxicity could cause excessive ROS generation, resulting in severe myocardial damage (He et al., 2018; Chen et al., 2019). Therefore, combinatorial treatment with Dox and antioxidants has been proposed to eliminate cardiotoxicity and endotheliotoxicity (Soultati et al., 2012; Wang et al., 2015; Akolkar et al., 2017; Abdel-Daim et al., 2017; He et al., 2018). Edaravone (Eda), a free radical scavenger, is the only neuroprotective agent for acute ischemic stroke used in Japan (Matsumoto et al., 2018). It captures and reduces excessive ROS, preventing brain damage. Studies have also confirmed that Eda inhibits ROS upstream, closes mitochondrial permeability transition pore (mPTP), prevents mitochondrial dysfunction, and in turn, potentially protects cells (Ikeda and Iwasaki, 2015; Matsumoto et al., 2018). Whether Eda can reduce the damage of VE caused by Dox toxicity is not clear.

Therefore, the aims of the study were to investigate by *in vivo* and *in vitro* (1) the subcellular and temporal characteristics of ROS generation in Dox toxicity-induced VE injury, (2) the role of ROS/endothelial nitric oxide synthase (eNOS)/nitric oxide (NO) pathway in Dox toxicity-induced VE injury, and (3) whether mitochondria are the target organelle of Dox-induced endotheliotoxicity.

## Materials and Methods

### Reagents, Cells, and Animals

Adenovirus pAD/eNOS was from GeneChem Co., Ltd (Shanghai, China). Dox, phenylephrine (PE), sodium nitroprusside (SNP), acetylcholine (Ach), Eda, N-nitro-l-arginine methylester (l-NAME), and ciclosporin A (CsA) were purchased from Sigma-Aldrich (St. Louis, MO, USA). Antibodies directed against eNOS, eNOS phospho-S1177, cytochrome C (*cyt c*), and β-actin were purchased from Abcam (Cambridge, UK). Horseradish peroxidase-conjugated IgG was from Jackson Immuno Research (West Grove, PA, USA).

Human umbilical vein endothelial cells (HUVECs) were purchased from the China infrastructure of cell line resources (Shanghai, China). Male C57BL/6J mice, 8-10 weeks old, weighing 20-22 g, were provided by the Animal Center of Nanchang University (Nanchang, China). All experimental protocols were performed following the National Institutes of Health (NIH) Guidelines for the Care and Use of Laboratory Animals (NIH Publication No. 85-23, revised 1996) and approved by the Ethics Committee of Nanchang University (Nanchang, China, No. 2018-0116).

### *In Vivo* Experiments

Mice were housed, two per cage, in a controlled environment at a temperature of 22°C and a humidity of 50%, a 12-hour light/dark cycle, and water was provided *ad libitum*.

#### Experimental Design

**Figure 1:**
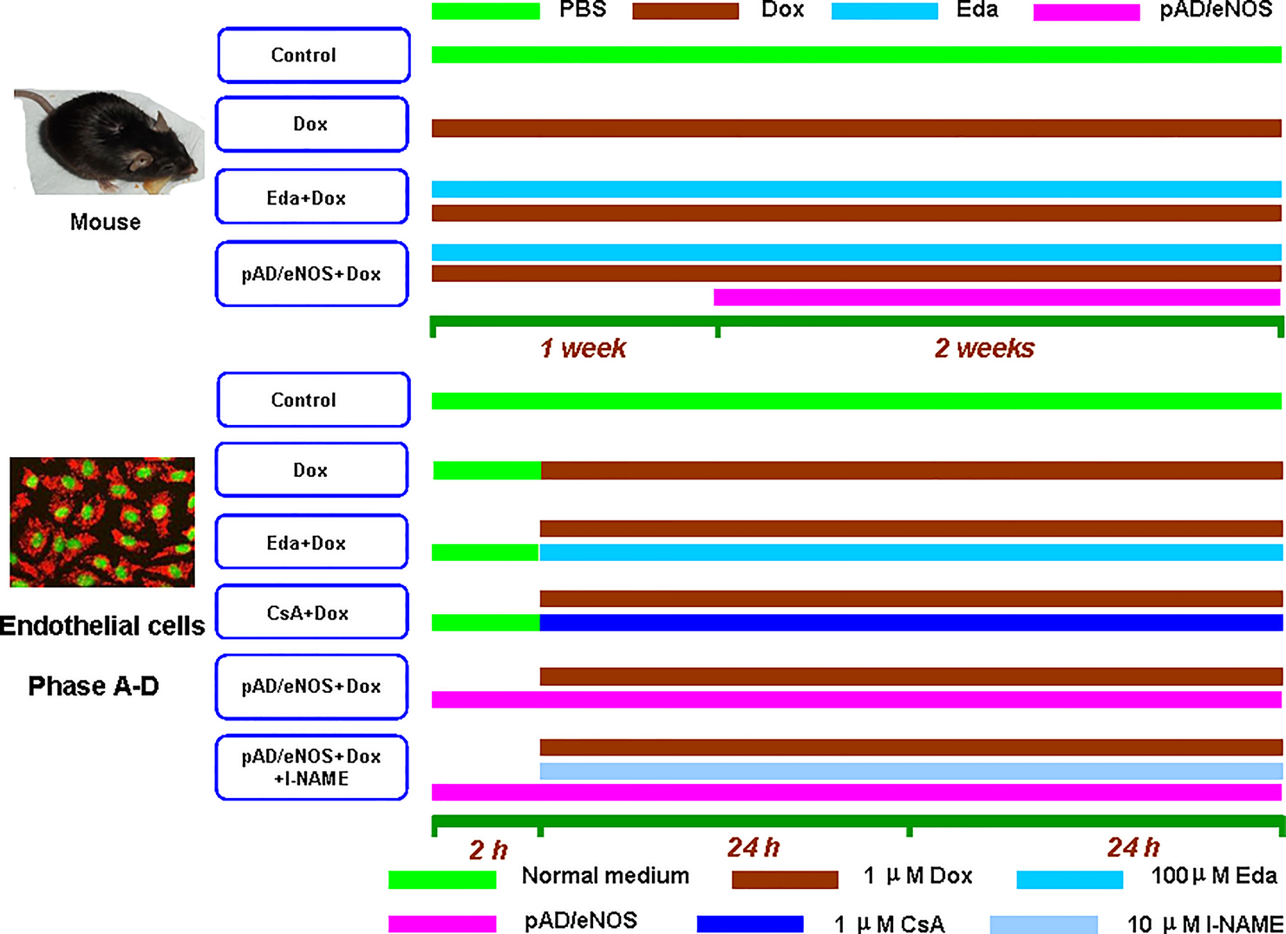
Schematic representation of the experimental design *in vivo* and *in vitro*. *In vivo*, 60 mice were randomly divided into four groups: thereinto three groups, Dox group, Eda+Dox group, and pAD/eNOS+Dox group, mice were intraperitoneal injected with six injections of 2.5 mg/kg Dox over 3 weeks for a cumulative dose of 15 mg/kg. Eda+Dox group: mice received Eda 10 mg/kg, daily for 3 weeks by intraperitoneal administration, an hour before Dox administration. pAD/eNOS+Dox group: mice were treated with the similar regime of Dox for one week, then pAD/eNOS adenovirus was injected. Control group: mice were given an equal volume of PBS using a similar regime as Eda+Dox group. *In vitro*, experimental groupings were as follows: Cells in control group were cultured under normal conditions during the entire experiment. Cells in Dox group was treated with 1 μM Dox for 48 h. Cells in eNOS^(+)^group were treated with pAD/eNOS for 2 h before Dox treatment. HUVECs in Eda/CsA group were treated similar to Dox group, and in addition cells were coincubated with 100 μM Eda/1 μM CsA for 48 h’ coincubation, respectively. Cells in eNOS^(+)^+l-NAME group was treated with the similar regime of eNOS^(+)^group, additionally 10 μM l-NAME for 48 h’ coincubation. HUVECs of these treatments were combined to form Phase A–D (See texts).

As shown in **Figure 1**, 60 mice were randomly divided into four different groups, 15 mice in each group: the Dox group: mice were intraperitoneally injected with six injections of 2.5 mg/kg Dox over 3 weeks for a cumulative dose of 15 mg/kg (He et al., 2018); the Eda+Dox group: mice administered 10 mg/kg Eda, once daily for 3 weeks *via* intraperitoneal administration (Ikeda and Iwasaki, 2015), an hour before Dox administration; the pAD/eNOS+Dox group: mice were treated with a regimen similar to the Dox group for one week, then pAD/eNOS adenovirus was injected into the body as follows. The control group: mice were given an equal volume of phosphate buffered saline (PBS) using a similar regime as the Eda+Dox group.

#### Gene Delivery *via* Tail Vein

An eNOS overexpression model was constructed in C57BL/6J mice *via* tail vein injection of recombinant adenovirus containing the gene for eNOS (Genbank ID 4846) as previously described (Chen et al., 2019). Briefly, pAD/eNOS adenovirus (2×10^11^ plaque-forming units/ml, 200 µl) were injected *via* the tail vein. At 2 weeks post injection, mice were sacrificed.

#### Collection of Blood and Tissue

At the end of the experiment, mice were weighed and anesthetized using intraperitoneal injection with ketamine (100–mg/kg) and xylazine (8 mg/kg). Then, blood was collected by cardiac puncture into heparinized capillary tubes and immediately centrifuged for 10 min at 3000 rpm for serum separation. Thoracic aorta rings were harvested in ice-cold physiologic saline solution (PSS: 0.288 g NaH_2_PO_4_, 1.802 g glucose, 0.44 g sodium pyruvate, 20.0 g BSA, 21.48 g NaCl, 0.875 g KCl, 0.7195 g MgSO_4_ 7H_2_0, 13.9 g MOPS sodium salt, and 0.185 g EDTA per liter solution at pH 7.4) and evaluated for vascular reactivity as described (Lee et al., 2018).

#### Determination of Activities of Serum Lactate Dehydrogenase (LDH) and Creatine Kinase (CK)

As a biomarker for tissue injury, the activities of serum LDH and CK were measured by a microplate reader (Bio-rad 680, Hercules, CA, USA) according to the specifications of the LDH assay kit and CK assay kit (Jiancheng, Nanjing, China).

#### Hematoxylin–Eosin Staining and TUNEL Assay

Freshly harvested thoracic aortas were fixed in 10% buffered formalin solution embedded in paraffin, and sectioned into 5-µm-thick sections that were mounted onto glass slides. To evaluate morphological changes, hematoxylin-eosin (H&E) staining was performed. To detect apoptosis, the terminal deoxynucleotidyl transferase mediated nick end labelling (TUNEL, Promega, Madison, WI, USA) staining method was performed according to the manufacturer’s guidelines (Qiao et al., 2016).

#### Vascular Reactivity

Vascular contractility and relaxation were determined as previously described (Lee et al., 2018; Wu et al., 2018). Briefly, thoracic aortas were placed in pressure myograph chambers (DMT Inc., Atlanta, GA, USA), containing warm PSS, cannulated and secured onto glass micropipettes, and equilibrated at an intraluminal pressure of 50 mmHg for 1 h at 37 C. First, we confirmed that arteries maintained constriction to PE (10^-10^-10^-4^ M) for the duration of the experiment until no spontaneous dilatation occurred during the constriction period (i.e., 5-12 min). Then, samples were constricted by increasing doses of PE (10^-6^ M, about EC_50_), immediately followed by a dose-response with an endothelium-dependent dilator ACh (10^-9^-10^-4^M). After a washout period and after pre-constriction to PE (10^-6^ M), a dose-response to the endothelium-independent dilator SNP (10^-10^-10^-4^ M) was performed. The percent of dilation was calculated based on the maximal luminal diameter of each artery.

#### Determination of NO Content

The NO content in mice’s serum or the culture medium was indirectly reflected by the contents of nitrite and nitrate (Wu et al., 2016). Nitrate is converted to nitrite by aspergillus nitrite reductase, and the total level of nitrite was measured using the Griess reagent (G4410, Sigma-Aldrich, St. Louis, MO, USA), for which the absorbance was determined at 540 nm. The NO content in samples was presented as the amounts of nitrite and nitrate (μM) per gram protein of serum or per liter of culture medium.

#### Western Blot Analysis

The protein from thoracic aortas samples, as well as the protein from HUVECs were extracted using a protein extraction kit (Applygen Technologies Inc, Beijing, China), respectively; 50 μg of protein was separated by denaturing SDS-polyacrylamide gel electrophoresis and transferred to polyvinylidene fluoride membranes. Membranes were then blocked with 5% skim milk, washed, incubated with primary antibodies directed against eNOS (1:1000), eNOS phospho-S1177(1: 1000), *cyt c* (1:1000), and β-actin (1:2000), then incubated with Horseradish peroxidase-conjugated secondary antibody. Subsequently, membranes were incubated with an enhanced chemiluminescence reagent for 2 min at 25°C, and protein bands were visualized using an enhanced chemiluminescence method and analyzed with Quantity One software (Bio-Rad, He et al., 2018).

### *In Vitro* Experiments

#### Endothelial Cell Culture and Adenovirus Transfection

HUVECs were cultivated in high-glucose Dulbecco’s modified Eagle medium (DMEM, Gibco-BRL, Grand Island, NY, USA) supplemented with 10% heat-inactivated fetal bovine serum (FBS, Gibco-BRL), penicillin (100 U/ml), and streptomycin (100 μg/ml), and cultured at 37°C in a humidified atmosphere at 5% CO_2_.

Constructs of pAD/eNOS (Genbank ID 4846) was transfected into HUVECs that were cultured in fresh DMEM medium and supplemented with 15% FBS. Transfection efficiency was roughly 85% after 48 h. Transfected cells were incubated at 37 °C, 95% O_2,_ and 5% CO_2_ for 2 h before being used for experiments.

#### Experimental Design (**Figure 1**)

##### Phase A

Firstly, we evaluated whether Dox could induce VE injury by multiple indexes.

HUVECs were randomly divided into four groups. Cells in the control group were cultured under normal conditions (37°C, 95% O_2,_ and 5% CO_2_) during the entire experiment. HUVECs in the Dox group was treated with 1 μM Dox for 48 h (He et al., 2018), whereas HUVECs in the eNOS^(+)^group were treated with pAD/eNOS for 2 h before Dox treatment. HUVECs in the Eda group were treated similar to the Dox group, but the cells were also coincubated with 100 μM Eda for 48 h (Masuda et al., 2016). At the end of the experiments, cell viability, apoptosis, the activities of LDH, and caspase-3 were determined.

##### Phase B

Next, we investigated whether Dox treated HUVECs could induce excess ROS generation. Furthermore, the subcellular and temporal characteristics of ROS generation were determined and the “ROS-induced ROS-release (RIRR)” was investigated.

HUVECs were randomly divided into four groups. Thereinto, the control, Dox, and Eda group was treated with the above Phase A. HUVECs in the CsA group were treated similar to the Dox group, but the cells were also coincubated with 1 μM CsA for 48 h (Teixeira et al., 2013). At the end of the experiments, cell viability and LDH activity were determined. At 0, 4, 8, 16, 24, and 48 h after the addition of Dox, intracellular/mitochondrial ROS generation of cells in each group were determined, respectively.

##### Phase C

We further investigated how excess ROS generation by induced Dox impaired VE and explored the role of eNOS/NO signaling pathway.

In brief, HUVECs were randomly divided into four groups. Thereinto, HUVECs in control, Dox, and eNOS^(+)^ group was treated with the above Phase A. HUVECs in the eNOS^(+)^+l-NAME group was treated similar to cells in the eNOS^(+)^group, but these cells were also coincubated for 48 h with 10 μM l-NAME (Ying et al., 2015).

At the end of the experiments, cell viability and LDH activity, NO content in the culture medium, the expression of eNOS, and eNOS phospho-S1177 in the lysate of HUVECs were determined.

##### Phase D

Finally, we studied how Dox damages the effector, mitochondria, and leads to its dysfunction.

In brief, HUVECs were randomly divided into four groups, namely, the control, Dox, Eda, and CsA group, which was treated with the above Phase B. At the end of the experiments, oxygen consumption rate (OCR), extracellular acidification rate (ECAR), mitochondrial membrane potential (MMP), mPTP opening, and *cyt c* release from mitochondria to cytoplasm in HUVECs were determined.

#### 3-(4,5-dimethylthiazol-2-yl)-5-(3-carboxymethoxyphenyl)-2-(4-Sulfophenyl)-2H-tetrazolium (MTS) Assay and Measurement of LDH Activity

HUVECs were plated in 96-well plates at a density of 1×10^4^ cells/well, incubated at 37°C with 20 μl MTS (5 mg/ml, Promega, Madison, WI, USA) in 100 μl of DMEM medium for 2 h. Next, the absorbance of each well was measured at 490 nm by a microplate reader (Bio-Rad680, Hercules, CA, USA). The absorbance was directly proportional to the number of live cells.

In HUVECs, LDH is an intracellular enzyme that is released into the culture medium upon cell damage (He et al., 2017). In this study, at the end of the experiment, supernatant was collected, and LDH activity was determined by a microplate reader (Bio-rad 680) according to the specifications of LDH assay kit (Jiancheng).

#### Caspase-3 Activity Assay

Caspase-3 activity was measured in the cytosolic fraction of isolated HUVECs, as described previously (He et al., 2017). Briefly, caspase-3 activity was determined by measuring the cleavage of a caspase-3-specific substrate[acetyl-Asp-Glu-Val-Asp (DEVD)-p-nitroanilide (pNA)(DEVD-pNA)] using a caspase-3 activity assay kit (R&D Systems, Minneapolis, MN, USA) according to the manufacturer’s instructions.

#### Assessment of Endothelial Apoptosis Using Annexin V-FITC and PI

Assessment of apoptosis of HUVECs was performed using an Annexin V-EGFP/PI apoptosis detection kit (BD Biosciences, San Diego, CA, USA). Annexin V-stained cells were analyzed using a Cytomics FC500 flow cytometer (Beckman Coulter, Brea, CA, USA) and DCF fluorescence was determined (He et al., 2018). The numbers of apoptotic cell were reflected by annexin V positive, PI negative population.

#### Measurement of Intracellular and Mitochondrial ROS

Levels of intracellular and mitochondrial ROS were measured using a DCFH-DA or mitoSOX probe as previously method (Zuo et al., 2018). In brief, at 0, 4, 8, 16, 24, and 48 h after corresponding treatment, cells were harvested, collected, and washed with serum-free DMEM media. Then, cells were mixed with serum-free media containing 10 μM DCFH-DA probe (Molecular Probes, Eugene, OR, USA) or 5 μM mitoSOX probe (Thermo Fisher Scientific, Waltham, MA, USA) and incubated at 37°C in the dark for 30 min with slight agitation every 5 min. Subsequently, cell pellets were collected, washed three times with PBS, and resuspended in 500 μl PBS for flow cytometry analysis (Cytomics FC500). The induced green fluorescence from 10,000 cells was documented at 488 or 510 nm. FlowJo software was used to analyze the average fluorescence intensity.

#### Evaluation of OCR and ECAR

Mitochondrial respiration is an indicator of both the functional bioenergetics capacity of mitochondria and overall cellular health (Zuo et al., 2018; Pan et al., 2018). In the study, we used an XFp Extracellular Flux Analyzer (Seahorse Biosciences, North Billerica, MA, USA) to evaluate the OCR. In brief, HUVECs were seeded in Seahorse XFp cell cultured miniplates at a density of 5,000 cells/well and subjected to corresponding treatment. The baseline rate was measured. The cells were subjected to the following three solutions: 10 μM oligomycin (complex V inhibitor), 2 μM carbonyl-cyanide-4-(trifluoromethoxy) phenyhydrazone (FCCP, permeabilized the inner mitochondrial membrane permeable for protons), and 0.5 μM of Rotenone (inhibitors of complex I and III) and antimycin A. A blank control was used to set the background. Data were expressed as pmol/min.

ECAR was determined by monitoring glycolytic function and was expressed as mph/min. the measurement procedure was similar to the measurement of OCR described above. after the measurement of basal ECAR, glucose solution (80 mm), oligomycin (5 mm), and 2-DG (100 mm) were added aequentially to determine glycolysis, glycolytic capacity, and the glycolytic reserve (Pan et al., 2018).

#### Assessment of MMP and mPTP Openness

Flow cytometry analysis was used to assess the loss of MMP by fluorescent indicator JC-1 (5,5’,6, 6’-tetrachloro-1,1’,3,3’-tetraethylbenzi-mida-zolo carbocyanine iodide, [Invitrogen, Carlsbad, CA, USA]). HUVECs were harvested and the cell suspension was incubated with JC-1 (200 μM) at 37°C for 20 min followed by washing twice with PBS to remove remaining reagents. Next, the fluorescence was measured by Cytomics FC500 flow cytometers with an initial excitation and emission wavelength (ex/em) at 530 and 580 nm (red), followed by ex/em at 485/530 nm (green), respectively. The ratio of red to green fluorescence intensity of cells reflected the level of MMP (He et al., 2017).

Mitochondria of HUVECs were isolated using a mitochondrial/cytosolic fractionation kit (Abcam), resuspended in swelling buffer (KCl 120 mM, Tris-HCl 10 mM, MOPS 20 mM, KH_2_PO_4_ 5 mM), and plated to a 96-well microtiter plate. The addition of 40 μl of CaCl_2_ solution (200 nM) to each well, acted as a stimulant of the opening of the mPTP and resulted in a steady decline in mitochondrial density. The absorbance at 520 nm was measured every minute until stable values were observed. To measure the extent of mPTP opening, the changes in absorbance were calculated (He et al., 2017).

## Statistical Analysis

Data were presented as the mean ± standard error of mean (S.E.M). The differences in the means between each group were tested by one-way analysis of variance (ANOVA) followed by Student-Newman-Keuls test (comparisons between multiple groups). *P* < 0.05 was considered statistically significant.

## Results

### Changes of Tissues Injury, Histopathology, Apoptosis, and Vascular Responsiveness on Dox Treated Mice

**Figure 2:**
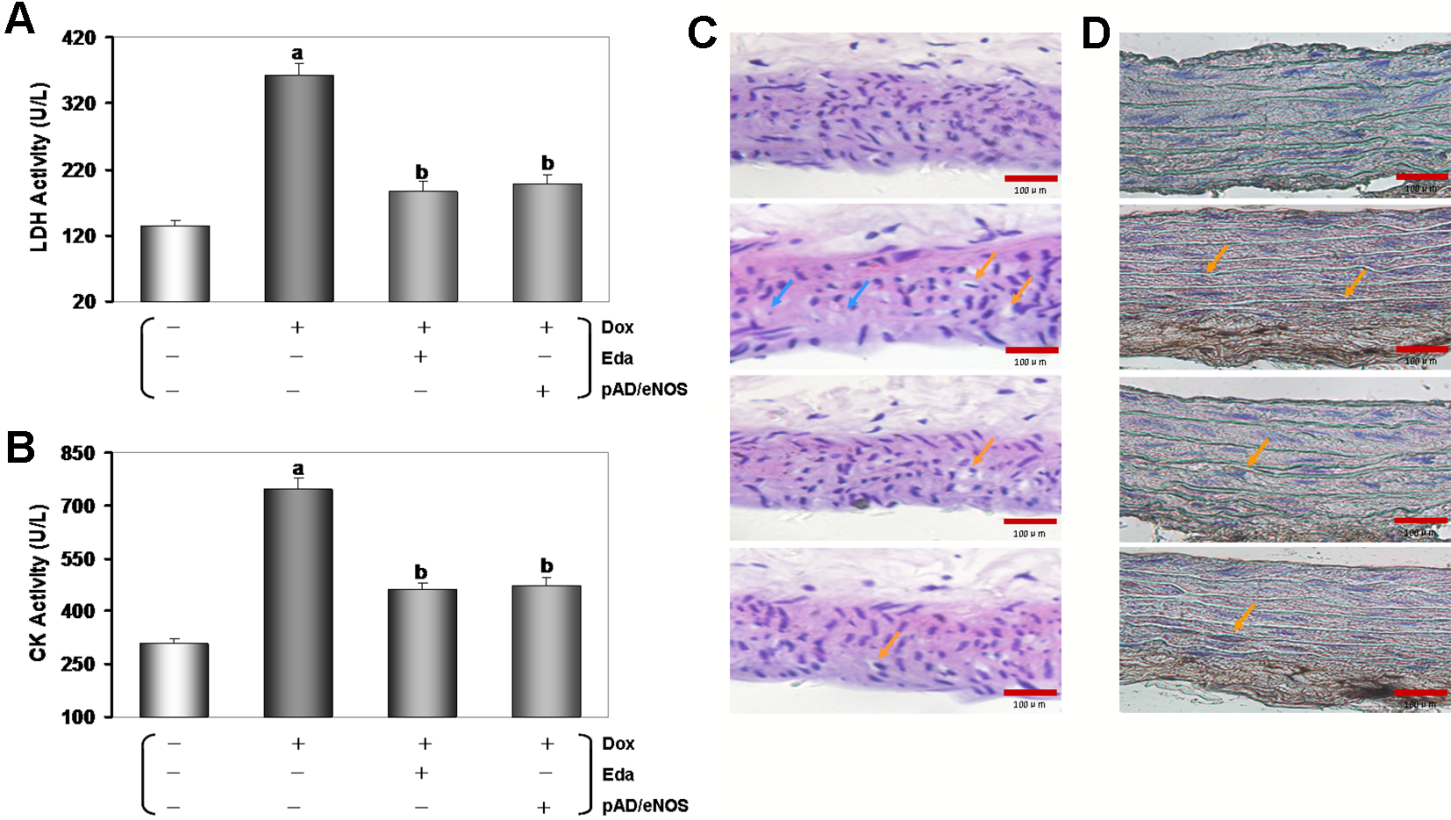
Changes of tissues injury, histopathological, and apoptosis in Dox-treated. **(A** and **B)** Activities of mice’s serum lactate dehydrogenase (LDH) and creatine kinase (CK). Data are presented as the mean ± S.E.M. for fifteen individual experiments. a: *P* < 0.01, vs. the control group; b: *P* < 0.01, vs. the Dox group. **(C)** hematoxylin-eosin (H&E) staining was performed for morphological analysis in the thoracic aortas tissue. Blue arrow: spotty necrosis; Orange arrow: hypertrophy of interstitial cells. **(D)** TUNEL staining was performed for morphological analysis in the thoracic aortas tissue. Orange arrow: TUNEL-positive cells.

As shown in **Figures 2A, B**, as expected, the activities of serum LDH and CK in the Dox group was significantly higher than that in the control group (*P* < 0.01), indicating that Dox had caused tissue and/or organ damage in mice. However, the activities of serum LDH and CK in the Eda+Dox group and the pAD/eNOS+Dox group were significantly improved (*P* < 0.01), suggesting that the treatment of Eda and pAD/eNOS could alleviate the abovementioned tissue and/or organ damage caused by Dox.

Thoracic aortas damage was confirmed by histopathological examination. As shown in **Figure 2C**, in the Dox group, some inflammatory changes, such as inflammatory infiltration, cell swelling, and interstitial cell hypertrophy, were found in the thoracic aortas tissue. However, the injury was significantly reduced in the Eda+Dox group and the pAD/eNOS+Dox group. Furthermore, the apoptosis of thoracic aortas tissue was assayed using TUNEL staining (**Figure 2D**). In microscopy, the Dox group clearly promoted apoptosis of thoracic aortas tissue when compared to that in the control group, as indicated by noticeable brown TUNEL-positive thoracic aortas tissue. However, the Eda+Dox group and the pAD/eNOS+Dox group significantly decreased in TUNEL-positive thoracic aortas tissue.

**Figure 3:**
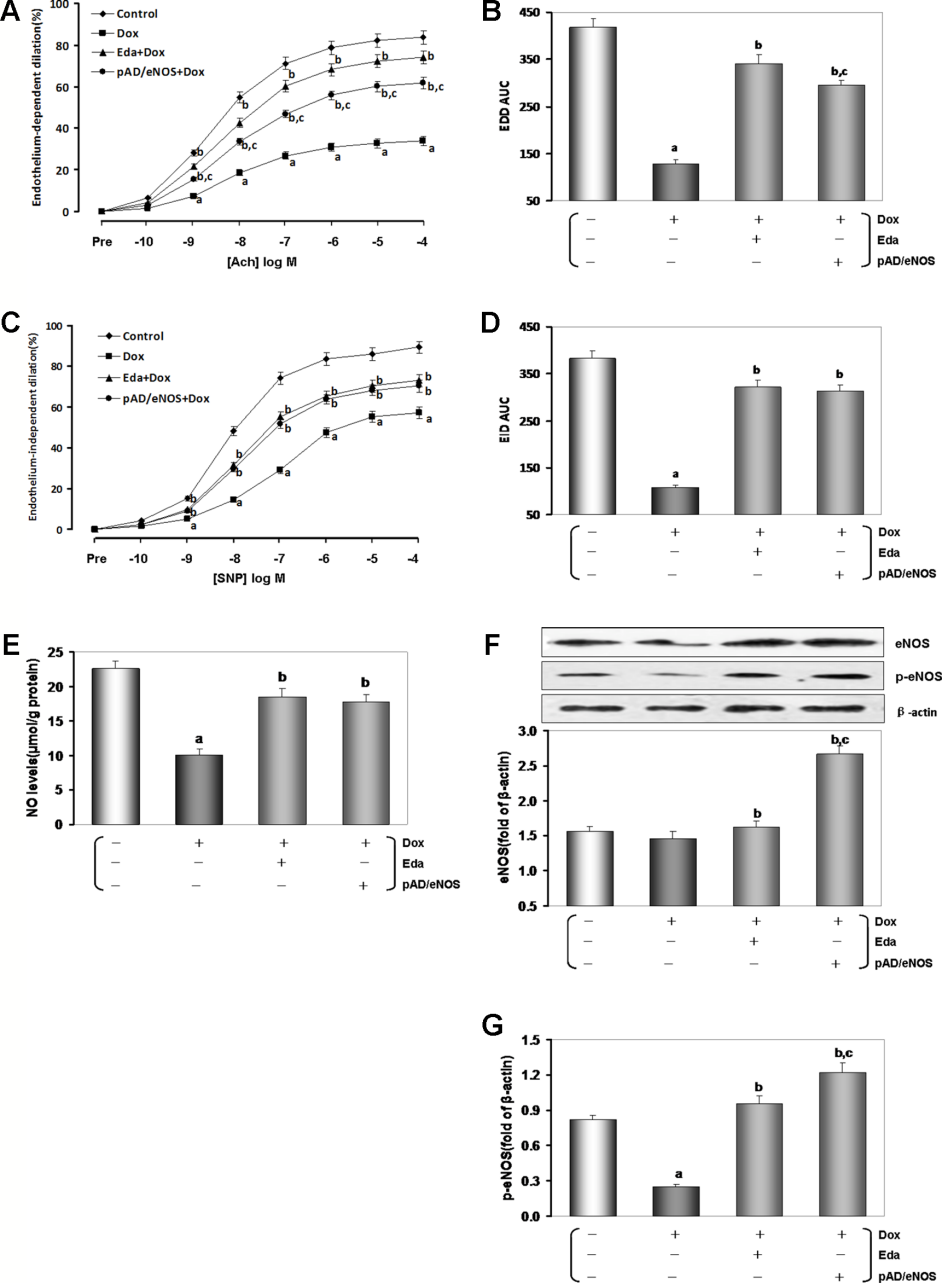
Changes of vascular responsiveness, eNOS/NO in serum, and aortic tissue homogenate in Dox-treated. **(A)** Endothelium-dependent dilation (EDD) of thoracic aortic strips. **(B)** Area under of the curve for EDD. **(C)** Endothelium-independent dilation (EID) of thoracic aortic strips. **(D)** Area under of the curve for EID. **(E)** Serum contents of NO. **(F)** eNOS expression in aortic tissue. **(G)** p-eNOS expression in aortic tissue. On **(F** and **G)**, from left to right, lane 1: control; lane 2: Dox; lane 3: Eda+Dox; lane 4: pAD/eNOS+Dox. Data are presented as the mean ± S.E.M. for fifteen individual experiments. a: *P* < 0.01, vs. the control group; b: *P* < 0.01, vs. the Dox group; c: *P* < 0.01, vs. the Eda+Dox group.

Generally, the control experiments of endothelium-dependent dilation (EDD) with Ach and endothelium-independent dilation (EID) with SNP are essential criteria for judging whether vascular endothelial cells normally function or not (Lee et al., 2018). As shown in **Figures 3A and B**, EDD in the Dox group was markedly impaired compared to the control group (*P* < 0.01), and area under the curve (AUC) of dose effect relationship decreased to 30.6% of the control group (*P* < 0.01). Eda and pAD/eNOS-treated improved EDD such that dilation was significantly increased at several doses of Ach (*P* < 0.01), and AUC also recovered to 81.6% and 70.6% of the control group, respectively (*P* < 0.01). Similarly, EID in the Dox group was significantly impaired (*P* < 0.01) and the AUC was 28.3% compared to the control group (*P* < 0.01, **Figures 3C, D**). Eda and pAD/eNOS treatment could also reverse the related changes (*P* < 0.01). This indicated that Dox toxicity could significantly damage VE. Of course, it also has considerable harm to vascular smooth muscle.

The above results indicated that Dox toxicity could cause VE damage in mice. Exogenous application of the free radical scavenger Eda or upregulation of eNOS expression could reverse the related damage.

### Changes of eNOS/NO in Dox Mice and Its Significance

In order to explore the possible mechanism of Dox toxicity to VE, we first examined the serum contents of NO in all mice. As illustrated in **Figure 3E**, the serum content of NO of the Dox group was much lower than that of the control group (*P* < 0.01). This change could be almost entirely counteracted by Eda and pAD/eNOS treatment (*P* < 0.01).

Furthermore, we detected the expression changes of eNOS and p-eNOS of the thoracic aortas tissue in all mice, respectively. As illustrated in **Figures 3F, G**, the aortic tissue in the Dox group, there was no significant change in the expression of eNOS (*P* > 0.05). However, the expression of p-eNOS was significantly downregulated (*P* < 0.01), with Eda and pAD/eNOS treatment, the expression of p-eNOS/eNOS were significantly upregulated (*P* < 0.01).

The above results indicated that Dox toxicity could inhibit the phosphorylation of eNOS, decrease NO synthesis, but Eda and pAD/eNOS treatment could upregulated the expression of eNOS, promote the phosphorylation of eNOS, and increase NO synthesis.

### Dox Toxicity Could Damage HUVECs Cells

**Figure 4:**
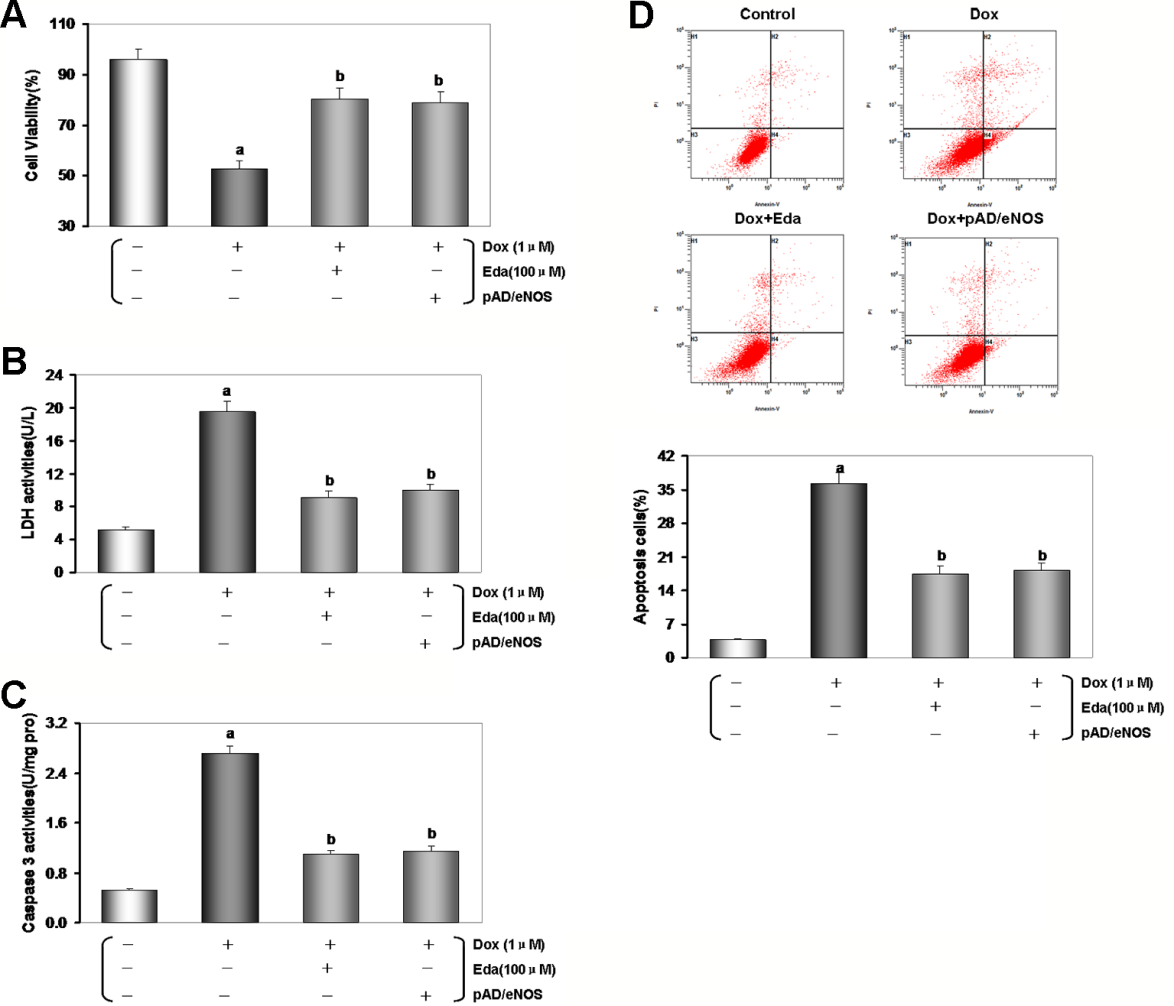
Dox toxicity could damage human umbilical vein endothelial cells (HUVECs). **(A)** Cell viability. **(B)** Lactate dehydrogenase (LDH) activity. **(C)** Histogram of the caspase-3 activity. **(D)** Flow cytometry dot plots (x-axis: annexin V-staining, y-axis: PI staining), and the quantitation of apoptotic cells. Data are presented as the mean ± S.E.M. for eight individual experiments. a: *P* < 0.01, vs. the control group; b: *P* < 0.01, vs. the Dox group.

In previous studies, we confirmed that 1 μM Dox for 24 h could induce myocardial cytotoxicity (He et al., 2018). Cell viability and LDH leakage generally serve as indexes of cell injury (Zhang et al., 2018). The MTS assay results showed that the viability of the Dox groups was significantly lower than that of the control group, and LDH activity of the Dox groups was significantly higher relative to that observed in the control group (*P* < 0.01, **Figures 4A, B**). After Eda and pAD/eNOS treatment, HUVECs injury was reversed, cell viability increased, and LDH activity decreased (*P* < 0.01).

However, cell viability and activity of LDH did no change using Eda alone, CsA alone, l-NAME alone, pAD/eNOS alone, and pAD/eNOS+l-NAME compared with the control group (*P* > 0.05, **Figures S1A, B** of **Supplementary Materials**).

As illustrated in **Figure 4C**, caspase-3 activity in the Dox group was significantly increased compared with the control group (*P* < 0.01), while Eda and pAD/eNOS treatment had a significant inhibition in caspase-3 activity compared with the Dox group (*P* < 0.01).

The degree of apoptosis in HUVECs was monitored through the quantitative analysis of Annexin V-EGFP/PI staining by flow cytometry analysis (He et al., 2017). As can be seen in **Figure 4D**, the ratio of apoptotic cells was notably higher in the Dox group than in the control group (*P* < 0.01). However, treatment with Eda and pAD/eNOS lowered the ratio of apoptotic cells (*P* < 0.01).

From the above results, we had confirmed that Dox toxicity could also cause HUVECs injury. However, exogenous supplementation of the free radical scavenger Eda or upregulation of eNOS expression can also effectively alleviate and reduce the damage of HUVECs caused by Dox toxicity.

### Dox Toxicity to HUVECs Could Induce Excessive ROS Generation, and the Role of RIRR

Previous, our studies show that excessive ROS generation by Dox toxicity trigger myocardial injury by mitochondria mediated (He et al., 2018; Chen et al., 2019), whether HUVECs toxicity by Dox will also cause excessive ROS generation and cause damage? First, we found that HUVECs were treated by 1 μM Dox after added 100 μM Eda (Masuda et al., 2016), a free radical scavenger, and 1 μM CsA (Teixeira et al., 2013), an mPTP closing agent, coincubation, cell viability increased and LDH activity decreased (*P* < 0.01, **Figures S2A, B** of **Supplementary Materials**). These results indicated that Eda and CsA alleviate caused HUVECs injury by Dox toxicity.

**Figure 5:**
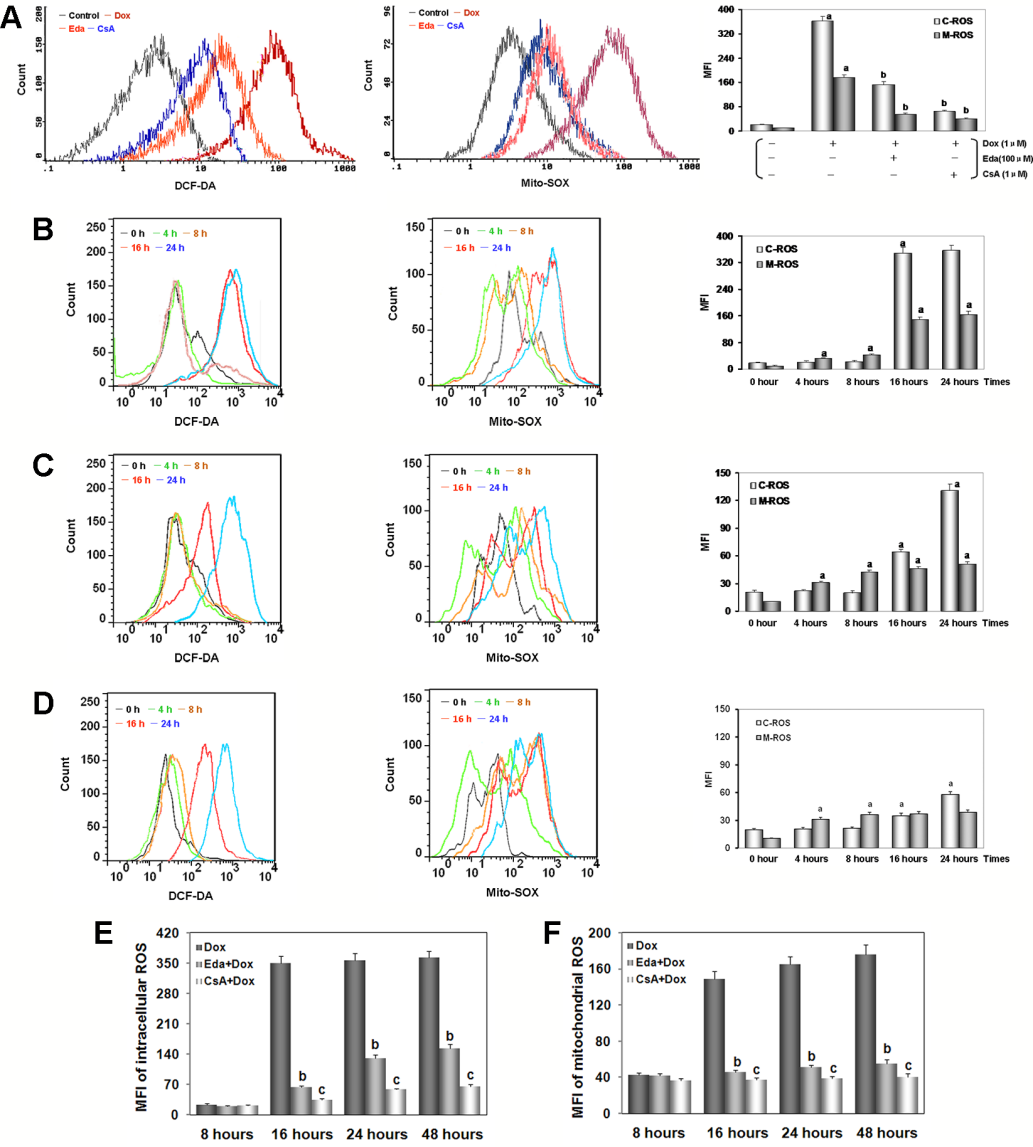
Dox toxicity in human umbilical vein endothelial cells (HUVECs) induced excess intracellular/mitochondrial reactive oxygen species (ROS) generation. **(A)** After addition of Dox for 48 h, intracellular/mitochondrial ROS generation of HUVECs after different treatments. **(B)** At various times after adding Dox, intracellular/mitochondrial ROS generation of HUVECs in Dox group. **(C)** At various times after adding Dox, intracellular/mitochondrial ROS generation of HUVECs in Eda group. **(D)** At various times after adding Dox, intracellular/mitochondrial ROS generation of HUVECs in CsA group. On **(A–D)**, left: intracellular ROS generation; middle: mitochondrial ROS generation; right: histogram of the intracellular/mitochondrial ROS generation. MFI: mean fluorescence intensity; C-ROS, intracellular ROS; M-ROS, mitochondrial ROS. **(E)** At various times after different treatments intracellular ROS generation of HUVECs. **(F)** At various times after different treatments mitochondrial ROS generation of HUVECs. Data are presented as the mean ± S.E.M for eight individual experiments. a: *P* < 0.01, vs. the control group; b: *P* < 0.01, vs. the Dox group; c: *P* < 0.01, vs. the Eda group.

After adding Dox for 48 h, the peak of intracellular/mitochondrial ROS in HUVECs was significantly moved to the right, indicating both significant increase in intracellular/mitochondrial ROS generation of the Dox group when compared with the control group (*P* < 0.01, **Figure 5A**). Moreover, adding Eda/CsA coincubation caused a significant shift of the peak of intracellular/mitochondrial ROS in HUVECs to the left, which indicated a significant decrease in intracellular/mitochondrial ROS generation (*P* < 0.01).

As illustrated in **Figure 5B**, in HUVECs after 1 μM Dox treatment, intracellular ROS generation stabilized at baseline between 4 and 8 h (*P* > 0.05), and suddenly increased more than fifteen times at 16 h (*P* < 0.01), and lasted until the experiments end of 48 h. At the same time, we found that after 1 μM Dox treatment for 4 h, mitochondrial ROS generation increased rapidly and persistently (*P* < 0.01), until ROS burst at 16 h increased more than fourteen times. These results suggested that: (1) HUVECs injury by Dox toxicity is indeed associated with excess ROS generation; (2) excess ROS generation originates in mitochondria rather than cytoplasm; (3) mitochondrial-dominated RIRR may be involved in the final ROS burst.

Interestingly, HUVECs were treated by 1 μM Dox after added 100 μM Eda coincubation, we found that ROS generation in both cytoplasm and mitochondria was similar to that in Dox treatment alone between 4 to 8 h, but increased in different degrees between 16 to 48 h, but the increase was significantly reduced, about one third or less of that in Dox treatment alone (**Figure 5C**). These results further confirm that excessive ROS is produced in mitochondria, because Eda can directly destroy free radical only in the cytoplasm, therefore, the extra ROS was not completely dealt with from the source, and the RIRR phenomenon could not be eliminated (**Figures 5E, F**).

Surprisingly, HUVECs was treated by 1 μM Dox, additionally 1 μM CsA coincubation, we found that throughout the experiment, the changes were similar to those in Eda group, except that the increase of ROS generation was even smaller between 16 and 48 h, which was only one-fifth or one-sixth of that in Dox treatment alone (**Figure 5D**). These results provided some convincing evidence that because CsA can shut down mPTP, mitochondrial ROS generation increased only slightly, cytoplasmic ROS generation increased only a limited extent, and ROS burst disappeared (**Figures 5E, F**). It was also confirmed that these phenomena are caused by excessive ROS generation in early cytoplasm or mitochondria, entering normal mitochondria and inducing RIRR. As a specific mPTP closing agent, CsA could ensure that even if a small amount of ROS is produced in the cytoplasm or mitochondria, it will not cause destructive damage to cells.

### Excessive ROS Generation by Dox Toxicity Impairs HUVECs, and the Possible Role of eNOS/NO Pathway

In the experiments, as mentioned above *in vivo*, we have revealed that eNOS/NO pathway may involve the damage of VE induced by Dox toxicity. In order to further confirm the mechanism of the related pathways, the specific inhibitor was used to explore HUVECs. However, we found that HUVECs were treated by 1 μM Dox after added pAD/eNOS and 10 μM l-NAME, a specific inhibitor of eNOS, coincubation, cell viability decreased and LDH activity increased (*P* < 0.01, **Figures S3A, B** of **Supplementary Materials**), these results indicated that upregulation of eNOS expression, but simultaneous inhibition of eNOS activity could aggravate HUVECs injury by Dox toxicity.

**Figure 6:**
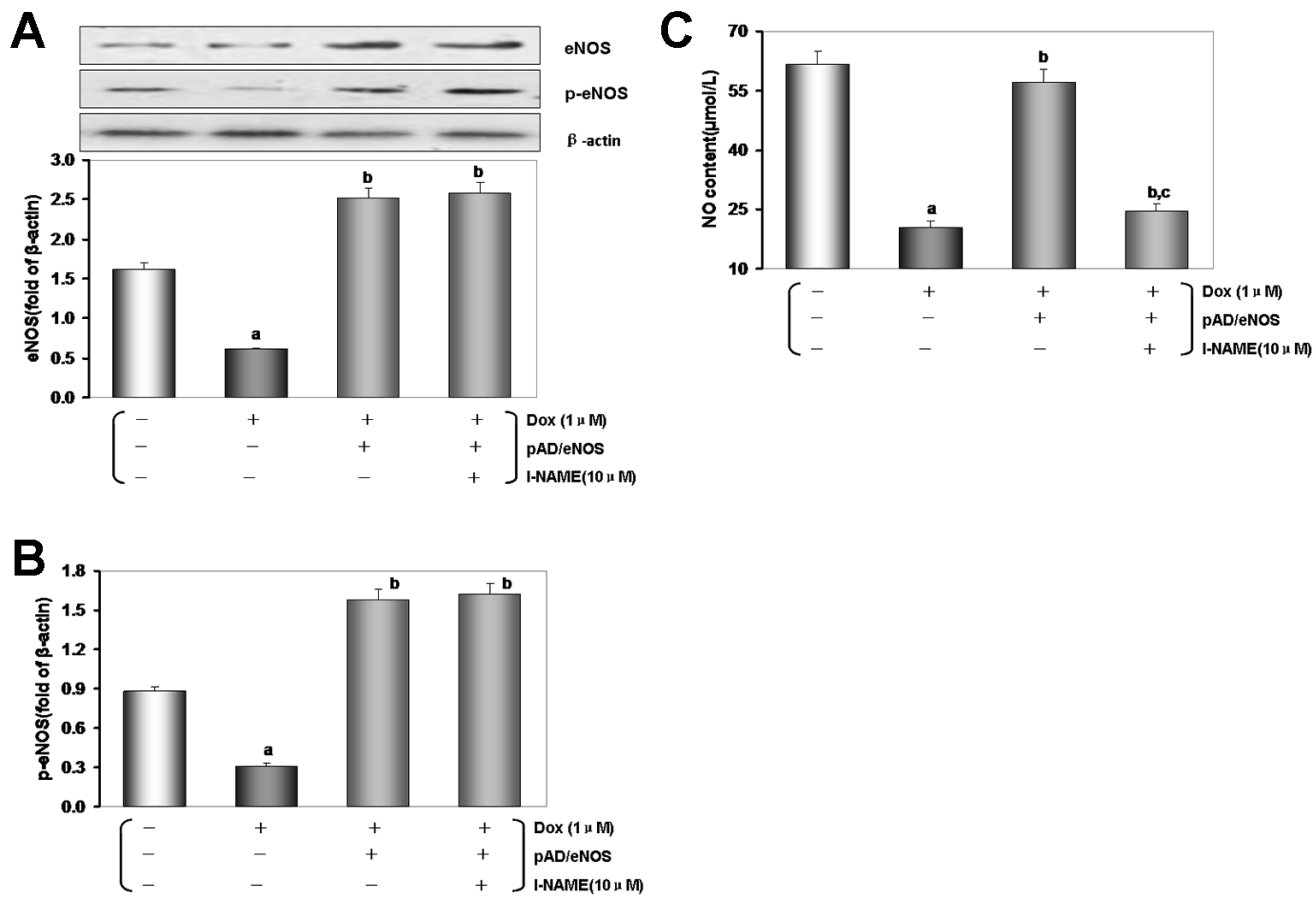
Excessive reactive oxygen species (ROS) generation by Dox toxicity impair human umbilical vein endothelial cells (HUVECs), and the possible role of eNOS/NO pathway. **(A)** eNOS expression in HUVECs. **(B)** p-eNOS expression in HUVECs. **(C)** The content of NO in culture medium. On **(A)** and **(B)**, from left to right, lane 1: control; lane 2: Dox; lane 3: pAD/eNOS+Dox; lane 4: pAD/eNOS+l-NAME+Dox. Data are presented as the mean ± S.E.M. for eight individual experiments. a: *P* < 0.01, vs. the control group; b: *P* < 0.01, vs. the Dox group; c: *P* < 0.01, vs. the pAD/eNOS+Dox group.

As illustrated in **Figures 6A, B**, in HUVECs lysate by Dox-treated, the expression of eNOS/p-eNOS reduced significantly than that of the control group (*P* < 0.01), but the effects were reversed by pAD/eNOS or pAD/eNOS and l-NAME (*P* < 0.01). Interestingly, content of NO in culture medium was significantly reduced after Dox-treated (**Figure 6C**, *P* < 0.01) and increased by pAD/eNOS; however, pAD/eNOS and l-NAME significantly decreased the NO content in culture medium (*P* < 0.01). These results indicated that Dox toxicity has a significant effect on the metabolism of eNOS and NO in VE.

### Dox Toxicity Damages the Effector, Mitochondria, How Does It Dysfunction

**Figure 7:**
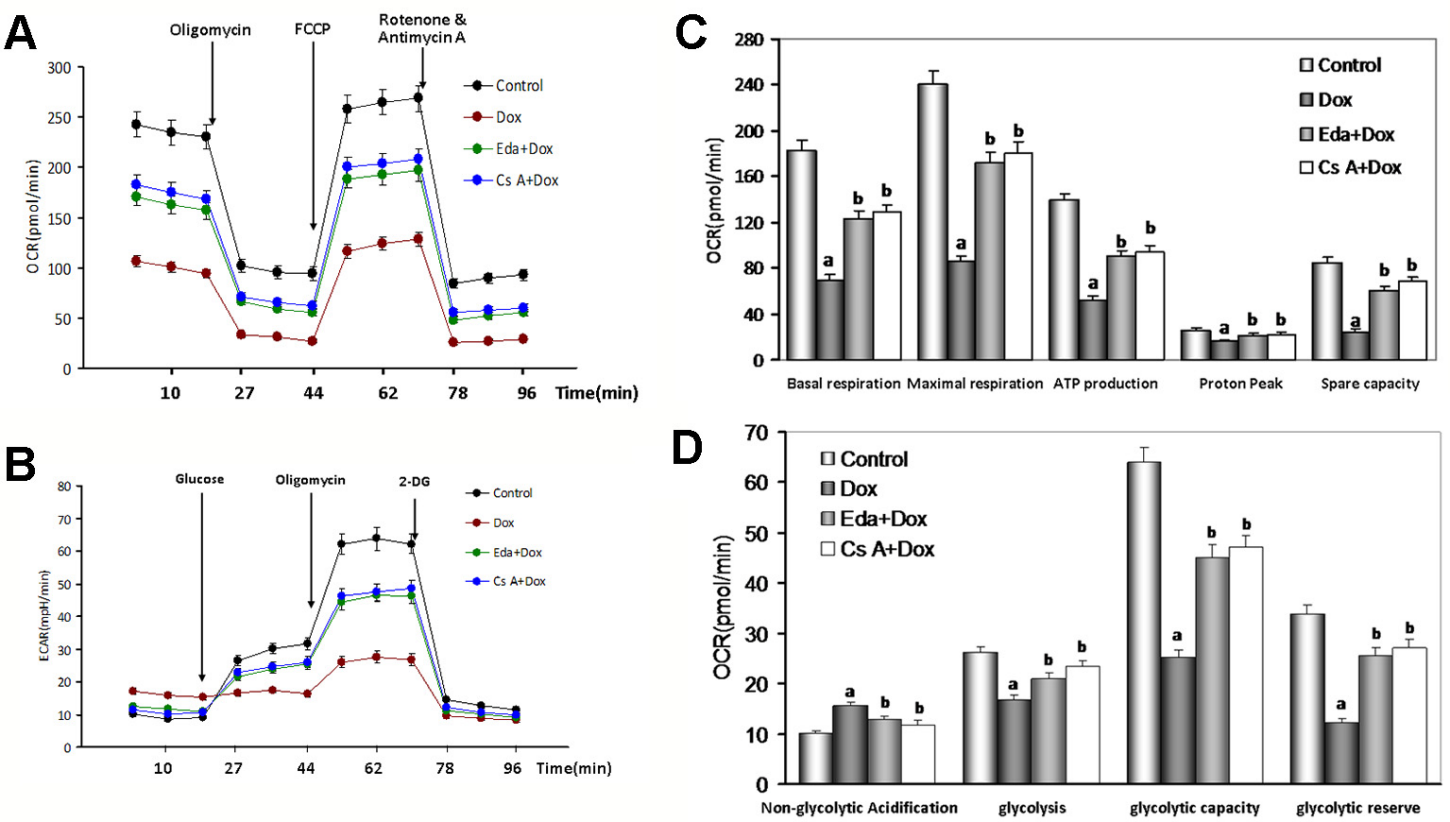
Changes of oxygen consumption rate (OCR) and extracellular acidification rate (ECAR) in human umbilical vein endothelial cells (HUVECs) by Dox toxicity injury. **(A)** Mitochondrial OCR curves obtained from different treatment. **(B)** Histogram of OCR important parameter from different treatment. **(C)** Mitochondrial ECAR curves obtained from different treatment. **(D)** Histogram of ECAR important parameter from different treatment. Data are presented as the mean ± S.E.M. for three individual experiments. a: *P* < 0.01, vs. the control group; b: *P* < 0.01, vs. the Dox group.

To explore the effects of Dox toxicity on mitochondrial respiration, OCR was measured using a Seahorse XF analyzer in HUVECs. The OCR of cell treated with 1 μM Dox-treated remained lower than the control group (*P* < 0.01, **Figure 7A**), in detail, as shown in **Figure 7B**, basal respiration, ATP production and proton peak, maximal respiration, and spare respiratory capacity, were all significantly lower in HUVECs by Dox- treated when compared to the control group (*P* < 0.01), therefore, when the addition of Eda and CsA, coincubation, the above changes significantly attenuated (*P* < 0.01).

ECAR was used to determine the changes in the glycolytic rate in HUVECs. As illustrated in **Figure 7C**, the ECAR of Dox-treated cells also remained lower than the control cells (*P* < 0.01), in detail, the basal rates of glycolysis and glycolytic capacity were all significantly lower in HUVECs by Dox-treated (*P* < 0.01), on the contrary, the non-glycolytic acidification had increased slightly. Similarly, the addition of Eda and CsA, coincubation, the above changes significantly attenuated (**Figure 7D**). Combined, these findings suggested that the energetic demand of HUVECs was decreased after Dox-treated because of mitochondrial damage. However, cotreatment by Eda and CsA significantly recovered mitochondrial respiration after Dox toxicity injury.

**Figure 8:**
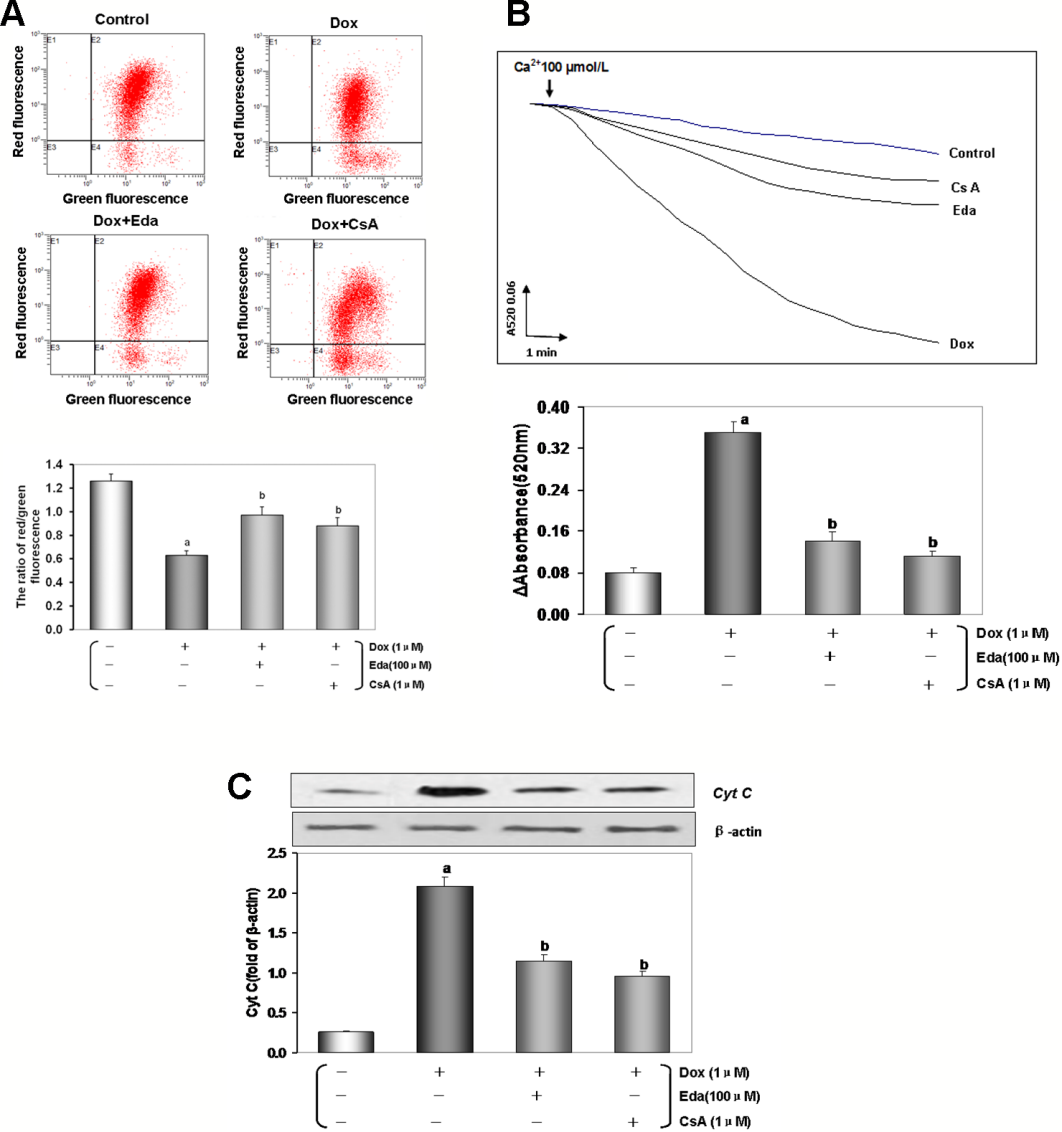
Dox toxicity could induce mitochondrial dysfunction in human umbilical vein endothelial cells (HUVECs). **(A)** Mitochondrial membrane potential (MMP) level was evaluated by JC-1. The ratio of red to green fluorescence intensity of cells reflected the level of MMP. **(B)** Ca^2+^-induced swelling of mitochondria was used to determine mPTP opening. The changes in absorbance at 520 nm were detected every 2 min. The data were accessed by the following equation: ΔOD = A520_0min_-A520_20min_. **(C)** Western blot analysis and histogram of *cyt c* expression in cytosol. From left to right, lane 1: control; lane 2: Dox; lane 3: Eda+Dox; lane 4: CsA+Dox. Data are presented as the mean ± S.E.M. for eight individual experiments. a: *P* < 0.01, vs. the control group; b: *P* < 0.01, vs. the Dox group.

Loss of MMP occurs in the early stages of apoptosis (Zorov et al., 2014). In live cells, JC-1 accumulates in the mitochondrial matrix and only exists in its monomeric form in apoptotic and dead cells because of the loss of MMP (He et al., 2017). As shown in **Figure 8A**, the reduction in red to green fluorescence ratio indicated a loss of MMP (*P* < 0.01) in the Dox group. Cotreatment by Eda and CsA resulted in a significant increase in MMP (*P* < 0.01).

mPTP opening is a major cause of cellular apoptosis. The status of mPTP opening was determined by Ca^2+^-induced swelling of mitochondrial (He et al., 2017). **Figure 8B** shows that when compared with the control group opening of the mPTP was induced after Dox-treated (*P* < 0.01). The cotreatment group by Eda and CsA showed a more gently downward trend than Dox-treated (*P* < 0.01).

As shown in **Figure 8C, D**ox toxicity injury resulted in a significant accumulation of *cyt c* in the cytosol (*P* < 0.01), and *cyt c* in the cytosol was significantly reduced when cells were cotreated with Eda and CsA (*P* < 0.01).

## Discussion

Dox is a chemotherapeutic drug to treat, including breast cancer, bladder cancer, Kaposi’s sarcoma, lymphoma, and acute lymphocytic leukemia. It is one of the most effective and safe medicines needed in the chemotherapy system of cancer (Bonadonna et al., 1969). However, in a dose-dependent manner, Dox could cause irreversible cardiomyopathy, resulting ultimately in heart failure. This cardiotoxicity of Dox is the leading cause of non-cancerous morbidity and mortality and has limited its clinical application (Singal and LIiskovic, 1998; Vejpongsa and Yeh, 2014). In addition to cardiotoxicity, many kinds of cytotoxicity caused by Dox have gradually attracted significant attention (Oliveira et al., 2014; Jacevic et al., 2017), especially endothelial dysfunction (Kotamraju et al., 2000; Wolf and Baynes, 2006; Wojcik et al., 2015). As a series of critical pathophysiological events of cardiovascular complications, endothelial dysfunction may cause hemorrhage, infarction, atherosclerosis, restenosis (Soultati et al., 2012; Wojcik et al., 2015).

Studies have shown that Dox could downregulate CX40 (Idris-Khodja et al., 2013), MnSOD expression and activity (Koka et al., 2010; Santos-Alves et al., 2019), regulate Bcl-2/Bax ratio (Durrant et al., 2015; You et al., 2019), promote the shift from topoisomerase II α to II β (Deng et al., 2014; Damiani et al., 2018), disturb microRNA, DNA methylation or protein acetylation (Nordgren et al., 2017; Ferreira et al., 2017), affect PARP-2/SIRT (Szántó et al., 2011), VEGF pathway (Chiusa et al., 2012), and interfere with tight junction formation between cardiac microvascular endothelial cells leading to increased permeability (Wilkinson et al., 2016). In the study, we proved *in vivo* that, after injected Dox with the classical protocol, the mice showed the LDH and CK activities in serum increased significantly (**Figures 2A, B**), the inflammatory changes were observed by histopathology (**Figure 2C**), which was noticeable as brown TUNEL-positive cells in microscopy (**Figure 2D**). The thoracic aortic strips’ EDD was significantly impaired (**Figure 3A**). Similarly, *in vitro,* we also confirmed that after exposure to 1 μM Dox for 24 h, HUVECs viability lowered, LDH activity in the culture medium, caspase-3 activity in the HUVECs homogenate and the ratio of apoptotic cells increased (**Figures 5** and **6**), that indicated Dox having induced the endotheliotoxicity.

More and more evidence demonstrate that in Dox-induced cytotoxicity ROS generation caused by triggers subsequent pathophysiological changes (Octavia et al., 2012; Soultati et al., 2012; Angsutararux et al., 2015; Wojcik et al., 2015; Cappetta et al., 2017; Renu et al., 2018). Studies have also shown that Dox accumulates through the reduction of the redox cycling in complex I of electron transport chain (ETC) in mitochondria, thereby increasing ROS generation (Renu et al., 2018). Mitochondrial NAD(P)H-dependent enzymes reduce Dox to corresponding semiquinone radicals, which undergo redox cycles to form superoxide radicals and hydrogen peroxide (Shabalala et al., 2017; Daiber et al., 2017a). In this study, HUVECs that were treated by Dox, both intracellular/mitochondrial ROS generation significantly increased (**Figure 5A**), thereby indicating that increased ROS was responsible for cell damage. Subsequently, we determined the intracellular/mitochondrial ROS generation at different time points and found a significant difference, that is, intracellular ROS generation significantly lagged behind mitochondria (**Figure 5B**). This result provides a direct experimental basis for mainstream literature reports (Renu et al., 2018).

Interestingly, at 16 h, we found that the trend of intracellular/mitochondrial ROS explosion was surprisingly consistent. The phenomenon is very similar to the RIRR hypothesis (Zorov et al., 2000; Zorov et al., 2014). This hypothesis suggests that when mitochondrial ROS increases, MMP becomes unstable, and mPTP opens continuously, then mitochondria swells and ruptures, irreversibly damage mitochondria. Therefore, ROS is released from its matrix into cytosol and absorbed rapidly by adjacent normal mitochondria, which induces similar changes in adjacent mitochondria, and cascade-like positive feedback amplifies, which ultimately leads to apoptosis (Brady et al., 2006; Zorov et al., 2014). Of course, the interaction between mitochondrial ROS with other sources of oxidative stress also plays an important role (Daiber, 2010; Schulz et al., 2014; Kröller-Schön et al., 2014). Surprisingly, we found that HUVECs treated with Dox plus Eda, a free radical scavenger (Masuda et al., 2016), could significantly delay or inhibit ROS burst from the source of RIRR, but could not completely cancel ROS burst (**Figures 5C, E, and F**). However, HUVECs treated with Dox plus CsA, an mPTP closing agent (Teixeira et al., 2013), could basically terminate ROS burst by closing mPTP at the end of RIRR (**Figures 5D–F**), therefore increasing cell viability and decreasing LDH activity (**Figures S2A, B**). The results also strongly suggest that free radical scavenger and mPTP closing reagent can effectively alleviate the endotheliotoxicity by Dox.

Studies have shown that cardiovascular homeostasis is heavily linked with NO signaling; vascular modulation warrants its transduction, which targets VE (Gajalakshmi et al., 2013). Early reports suggested that Dox-induced endotheliotoxicity has been linked to excessive ROS generation and increased redox-cycling of the Dox-semiquinone radical, which lead to increased intracellular oxidant stress, disrupt of NO/superoxide balance, and alter the endothelial elasticitelasticity (Vásquez-Vivar et al., 1997; Kalivendi et al., 2001). In other words, the reduction in eNOS leads to the decreased bioavailability of NO in endothelial cells which contributes to endothelial dysfunction. Thus, the structural and functional integrity of eNOS is critical (Sessa et al., 1995). The multistep controls of eNOS are (1) expression of eNOS, (2) eNOS phosphorylation, and (3) sub-cellular trafficking and localization of eNOS (Sessa, 1994). Generally, phosphorylation and dephosphorylation are the key ways to regulate the biological activity of functional proteins. Ser-1177 on eNOS, phosphorylation of a COOH-terminal Akt/PKB-dependent phosphorylation site, will displace the regulatory COOH-terminal tail to relieve repression of NO synthesis (Balligand et al., 2009). Recently, researchers have a further understanding of the effects of Dox on the phosphorylation of eNOS: Dox can further affect the formation of monomer/dimer ratio of eNOS by influencing BRCA1, ALDH2, NOX4, etc., or Ang II receptor, regulating Akt and other signal pathways, which can result in eNOS uncoupling, inhibit the phosphorylation of eNOS and catalyze the biological synthesis of NO (Singh et al., 2013; Ge et al., 2016; Octavia et al., 2017; Toedebusch et al., 2018; Mu et al., 2019). However, the redox crosstalk of superoxide/hydrogen peroxide produced by mitochondria with other ROS producing enzymes such as NADPH oxidases are also of outstanding importance, which further induces eNOS uncoupling (Deng et al., 2007; Daiber, 2010; Kröller-Schön et al., 2014; Daiber et al., 2017a).

At present, strategies to increase the bioavailability of NO in VE have been proven to contribute to endothelial protection. For example, ginsenoside Rg3 (Wang et al., 2015), Ursolic acid (Mu et al., 2019), vanilloid 1 (Ge et al., 2016), fidarestat (Sonowal et al., 2018), Folic acid (Octavia et al., 2017), zofenoprilat (Monti et al., 2013) can upregulate eNOS expression and phosphorylation, enhance its activity, ultimately remove NO deficit and restore the NO bioavailability. *In vivo* and *in vitro,* our data after Dox-treated have shown that with the increase of ROS generation, and endothelial dysfunction, eNOS expression decreased, especially eNOS phosphory-lation, and thereby NO content decreased. However, exogenous supplements Eda, a free radical scavenger (Masuda et al., 2016), or pAD/eNOS treatment, which could upregulated the expression of eNOS, promote eNOS phosphorylation, increase NO synthesis (**Figures 3** and **6**), and improve endothelial function.

Mitochondria are multifunctional organelles and can actively or passively drive cellular dysfunction or demise (Lemasters et al., 1998). Indeed, the structural and functional integrity is fundamental to cellular life. Apoptosis, degeneration, and necrosis often cooccur in VE injury (Tang et al., 2014; Szewczyk et al., 2015). It is well known that mitochondria are essential to target organelles for Dox-induced cardiomyopathy, and the improvement of mitochondrial dysfunction can prevent myocardial damage (Octavia et al., 2012). It has also been proved by our previous work (He et al., 2018; Chen et al., 2019). Is the target organelle of Dox induced endotheliotoxicity consistent with that of cardiotoxicity? In the study, we found that in HUVECs that were treated with Dox for 48 h, the mitochondrial function was marked impaired, reflecting in that mitochondrial respiration and glycolytic function (the abilities of oxidative phosphorylation and ATP production) was weakened significantly (**Figure 7**), impeded MMP, mediated mitochondrial swelling, opened mPTP, and released *cyt c* from mitochondria into cytosol. Of course, adding Eda or CsA caused the above-mentioned mitochondrial function to recover and improve significantly (**Figure 8**). These results indicated that mitochondria are the target organelles of Dox-induced endotheliotoxicity, and may also be the molecular targets for the prevention and treatment of the corresponding injury.

## Limitation of the Study

In the study, we used DCF-DA and mitoSOX staining which are not optimal for ROS detection since they are not specific for a certain species (Daiber et al., 2017b). It is also not ideal to measure eNOS activity with nitrite (Dobi et al., 2019), because it is easy to be interfered by diet, etc., which may limit the universality of the results obtained.

## Conclusions

**Figure 9:**
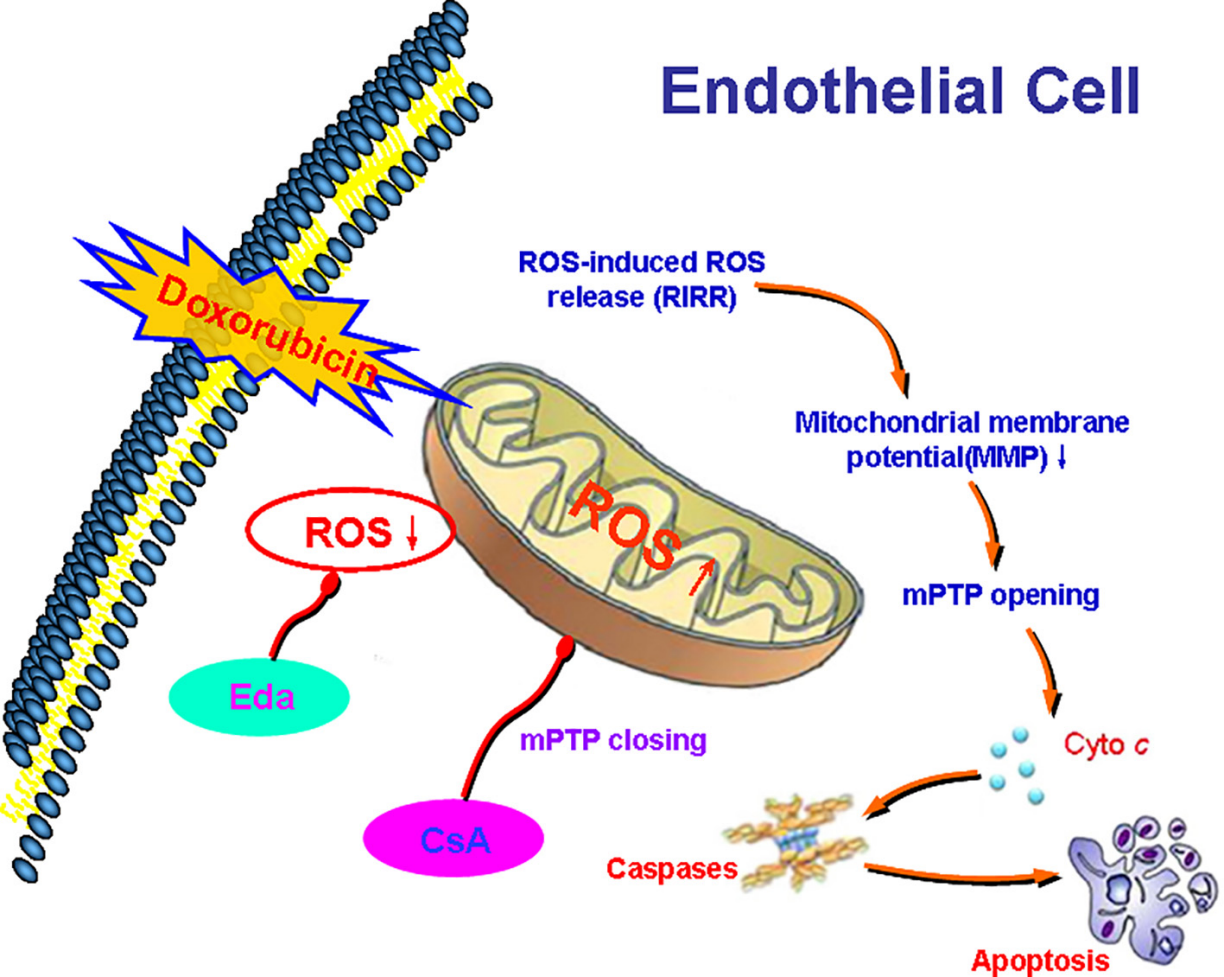
Diagram showing the possible mechanism of Dox toxicity injury to vascular endothelium (VE). Dox produces excess reactive oxygen species (ROS) in mitochondria, thereby weakening mitochondrial membrane potential (MMP), opening mitochondrial permeability transition pore (mPTP), activating ROS-induced ROS-release (RIRR) mechanism, inducing ROS burst, leading to mitochondrial dysfunction, which in turn damages VE. Therefore, interrupting any step of the abovementioned cycles can end the related vicious cycle and prevent the occurrence and development of injury.

We explored ROS as the core and outline the possible mechanism of Dox-induced endotheliotoxicity (**Figure 9**). Dox produces excess ROS in mitochondria, thereby weakening MMP, opening mPTP, activating RIRR mechanism, inducing ROS burst, and leading to mitochondrial dysfunction, which in turn damages VE. Therefore, interrupting any step of the cycles, as mentioned earlier, can end the related vicious cycle and prevent the occurrence and development of injury.

## Data Availability Statement

All datasets generated for this study are included in the article/**Supplementary Material**.

## Ethics Statement

The animal study was reviewed and approved by the Ethics Committee of Nanchang University (No. 2018-0116)

## Author Contributions

MH and HH conceived and designed the experiments. HH, LW, YQ, QZ, HL, and SC performed the experiments. DY and QH analyzed the data. HH, DY, and QH contributed reagents/materials/analysis tools. HH, QH, and MH wrote the paper.

## Funding

This research was supported by grants from the National Natural Science Foundation of China (№: 21467017, 81673431, 81660538, 81803534) and Jiangxi Applied Research and Cultivation Program (20181BBG78059).

## Conflict of Interest

The authors declare that the research was conducted in the absence of any commercial or financial relationships that could be construed as a potential conflict of interest.

## Abbreviations

Ach, acetylcholine; AUC, area under the curve; CK, creatine kinase; CsA, ciclosporin A; *cyt c*, cytochrome C; DCFH-DA, 6-carboxy-2`-7`- Dichlorodihydro-flurescein diacetate; DMEM, Dulbecco’s modified Eagle medium; Dox, doxorubicin; ECAR, extracelluar acidification rate; Eda, edaravone; EDD, endothelium-dependent dilation; EID, endothelium-independent dilation; eNOS, endothelial nitric oxide synthase; H&E, hematoxylin-eosin staining; HUVECs, human umbilical vein endothelial cells; LDH, lactate dehydrogenase; l-NAME, N-nitro-l-arginine methyl ester; MMP, mitochondrial membrane potential; mPTP, mitochonondria permeability transition pore; NO, nitric oxide; OCR, oxygen consumption rate; PE, phenylephrine; PSS, physiologic saline solution; RIRR, ROS-induced ROS release; ROS, reactive oxygen species; SNP, sodium nitroprusside; TUNEL, terminal deoxynucleotidyl transferase d UTP nick-end labeling; VE, vascular endothelium.
