# Supplementary material for: Doxorubicin Induces Endotheliotoxicity and Mitochondrial Dysfunction via ROS/eNOS/NO Pathway
Source: Front Pharmacol. 2020 Jan 10;10:1531. doi: 10.3389/fphar.2019.01531 (PMC6965327; doi:10.3389/fphar.2019.01531)
Supplement: Supplementary file 1 [file DataSheet_1.pdf]

Supplementary materials

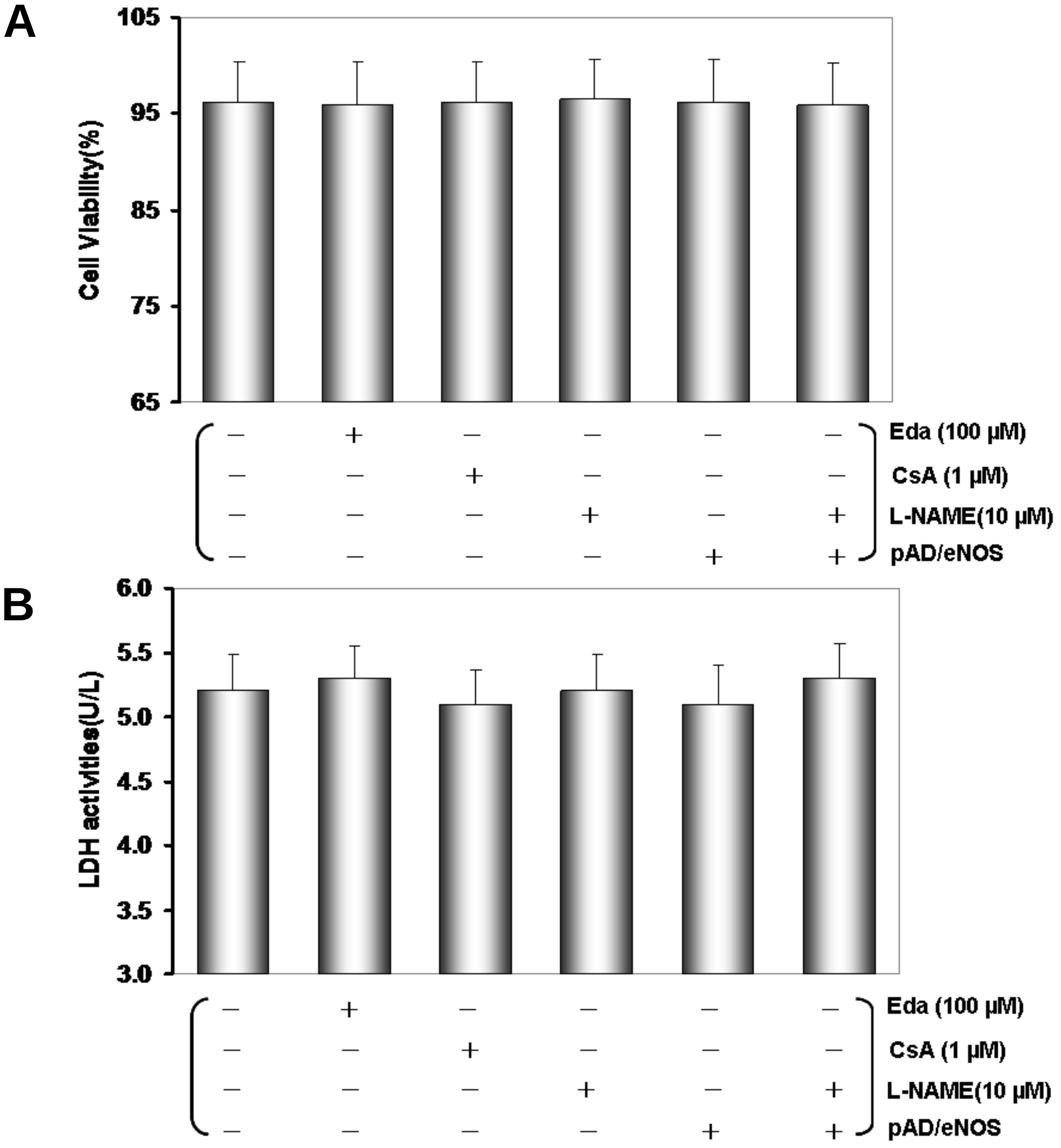

Figure S1. Effects of Eda/CsA, or upregulated eNOS expression, or upregulated eNOS expression added l-NAME on cell viability and LDH activity of HUVECs**.** Cell viability and LDH activity did not change by using Eda alone (100 μM), CsA alone (1 μM), or upregulated eNOS expression alone, or upregulated eNOS expression added l-NAME (10 μM) when compared with the control group (P>0.05). (A) Histogram of cell viability. (B) Histogram of LDH activity. Data are presented as the mean ± S.E.M. for eight individual experiments.

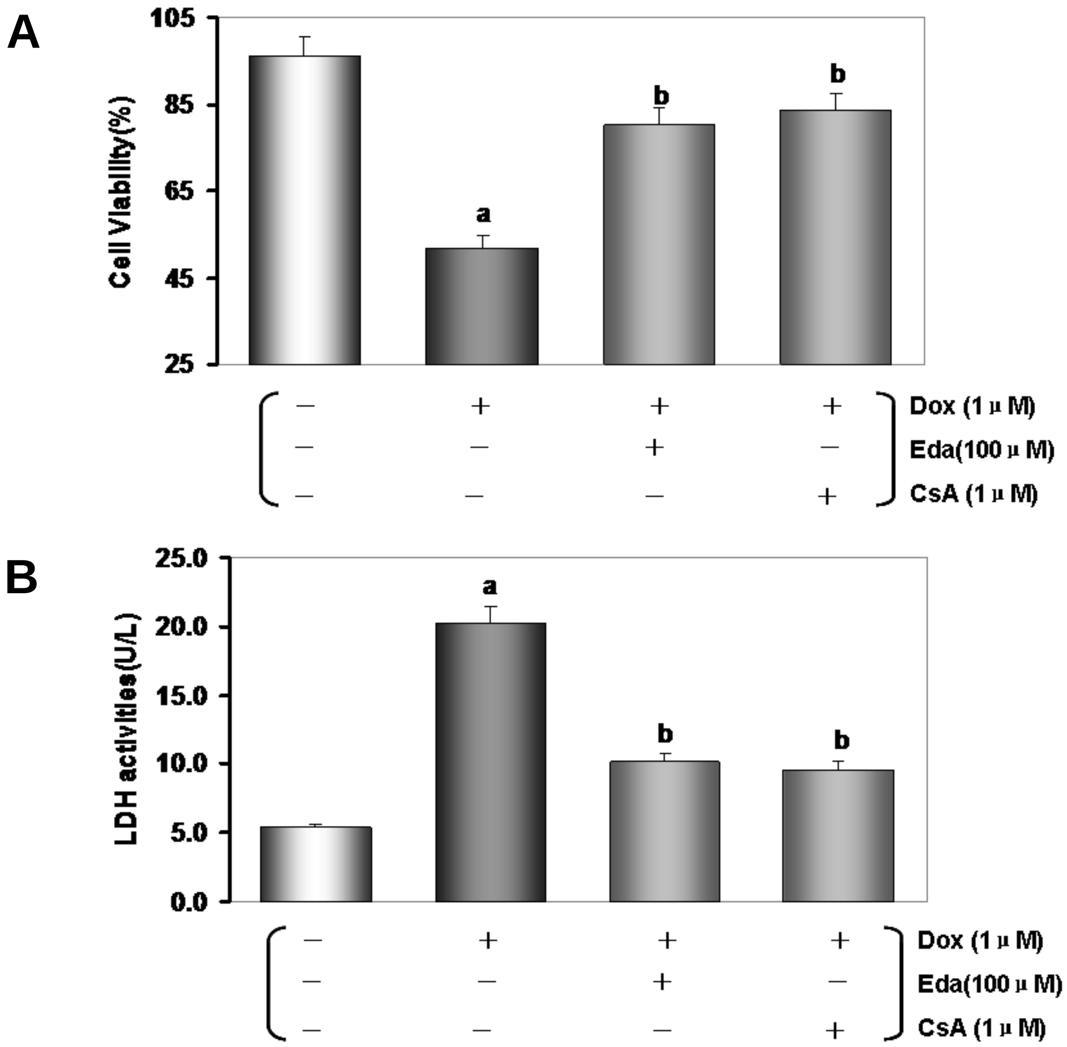

Figure S2. Effects of Eda/CsA on cell viability and LDH activity of HUVECs injuried by 1 μM Dox**.** Eda/CsA with 1 μM Dox co-treat HUVECs, cell viability increased and LDH activity decreased. (A) Histogram of cell viability. (B) Histogram of LDH activity. Data are presented as the mean ± S.E.M. for eight individual experiments. a: *P*<0.01, vs. the control group; b: *P*<0.01, vs. the Dox group.

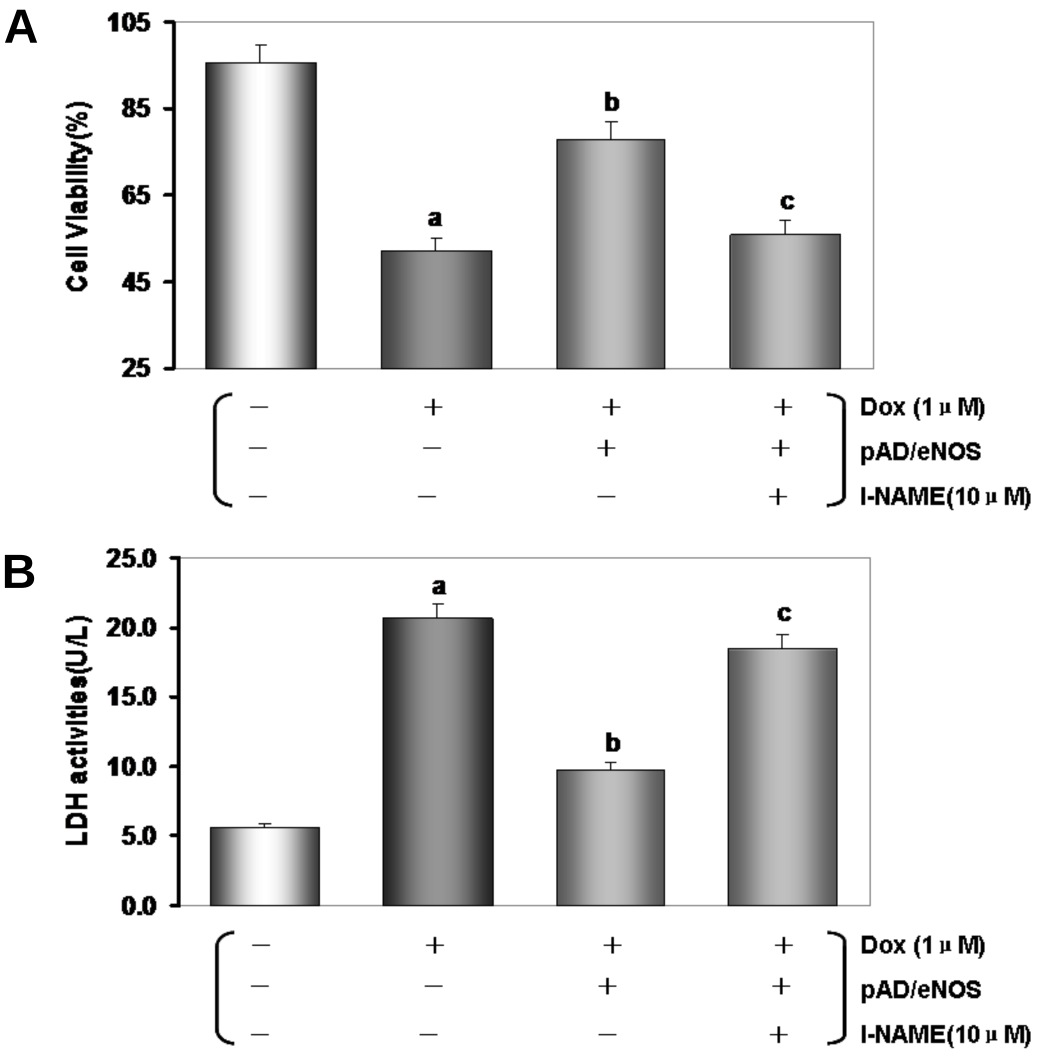

Figure S3. Effects of upregulated eNOS expression, or upregulated eNOS expression added l-NAME on cell viability and LDH activity of HUVECs injuried by 1 μM Dox**.** Upregulated eNOS expression protected HUVECs against Dox injury, but added l-NAME reversed the effects. (A) Histogram of cell viability. (B) Histogram of LDH activity. Data are presented as the mean ± S.E.M. for eight individual experiments. a: $P<0.01$, vs. the control group; b: $P<0.01$, vs. the Dox group; c: $P<0.01$, vs. the Dox+pAD/eNOS group.
